# Increased activation and cytokine secretion in B cells stimulated with leptin in aged humans

**DOI:** 10.1186/1742-4933-10-3

**Published:** 2013-01-23

**Authors:** Sudhir Gupta, Sudhanshu Agrawal, Sastry Gollapudi

**Affiliations:** 1 Programs in Primary Immunodeficiency and Aging, Division of Basic and Clinical Immunology, University of California, Irvine, CA, USA; 2 Medical Sciences I, C-240, University of California, Irvine, CA, 92697, USA

**Keywords:** TNF-α, IL-6, IL-10, B cells

## Abstract

Aging is associated chronic inflammation and autoimmunity, and increased levels of leptin. Increased levels of leptin are associated with inflammation and autoimmunity. We have recently reported that leptin activates B cells to induce secretion of proinflammatory and anti-inflammatory cytokines. Role of B cells and leptin in inflammation associated with aging has not been explored. In this study we demonstrate that leptin activates and induces significantly greater amount of IL-6, TNF-α, and IL-10 by B cells from aged humans as compared to young controls. This is associated with increased leptin-induced phosphorylation of STAT3 (signal transducer and activator of transcription-3) in B cells from aged humans as compared to young subjects. These data suggest that leptin-induced B cell-derived proinflammatory cytokines may play a role in chronic inflammation associated with human aging.

## Background

Leptin, one of adipokines induces secretion of a number of proinflammatory cytokines, including IL-6, IL-1, and TNF-α [1-5]. Serum levels of leptin,TNF-α, IL-6, and other proinflammatory cytokines are increased during human aging and age-associated inflammatory disorders [6-15]. However, the cellular source of these cytokines in aging is poorly understood. These cytokines are produced by T cells, macrophages, and B cells. However, T cell-derived and macrophage-derived proinflammatory cytokines are decreased in aging [16-20]. Therefore, B cells are likely the source of increased proinflammatory cytokines in aging. B cell derived inflammatory cytokines have not been studied in aging. During aging adipose tissue is increased in the lymph nodes and the bone marrow, which may result in leptin levels that may be 10 to 100 fold greater than those in plasma. In addition to their well-defined role in antibody production, B cells may also regulate immune responses to microbes and participate in inflammation through production of cytokines, chemokines, and growth factors [21-24]. Human B cells have shown to produce cytokines, including proinflammatory cytokines IL-6, TNF-α, and immunoregulatory and anti-inflammatory cytokines, such TGF-β, and IL-10 [21-24]. We have recently reported that leptin activates human B cells to induce secretion of IL-6 and TNF-α and IL-10, and this effect of leptin on B cells is mediated via JAK2/STAT3 and p38MAPK/ERK1/2 signaling pathways [25].

In this study, we show that leptin activates B cells and B cell subsets from aged subjects to a significantly greater extent, and induces significantly higher levels of secretion of IL-6, TNF-α, and IL-10 as compared to B cells and B cell subsets from young subjects. This appears to be due to an increased leptin-induced phosphorylation of STAT-3 in B cells from aged as compared to young controls.

## Results

### Leptin induces greater activation of B cells from aged as compared to young humans

**Figure 1:**
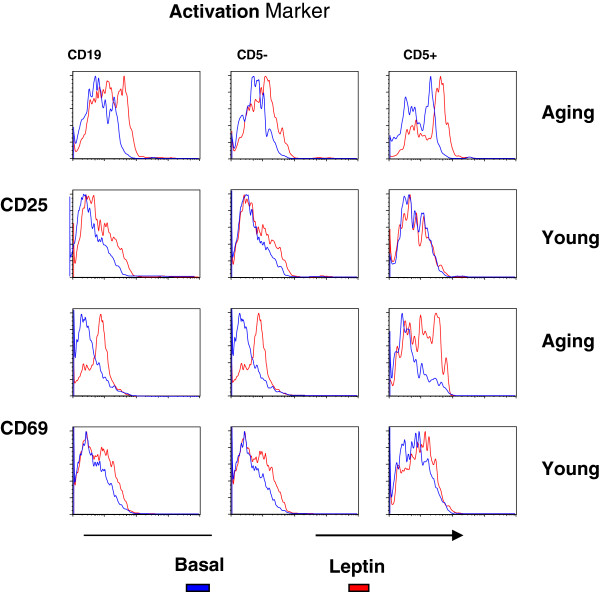
**Effect of leptin on activation of B cells and B cell subsets.** Purified B cells from young and aged subjects were stimulated with leptin (50 ng/ml) for 24 hours and expression of CD25 and CD69 as activation markers, using specific antibodies and isotype controls. Blue lines are for unactivated B cells, and red line for leptin-activated B cells.

Purified B cells were activated with 50 ng/ml of leptin (optimum concentration-reference# 25) for 24 hours. Cells were washed and stained with anti-CD19 antibody, anti-CD5 antibody, and antibodies directed against CD25, and CD69 as activation markers. Figure 1 shows a representative cytofluorograph. In aging, leptin induced (red line) greater activation (increased expression of CD25 and CD69) over the baseline levels (blue control) as compared to young control. Furthermore, increased activation in aging was observed in both CD5+ and CD5- B cells. Tables 1 and 2 show data from 10 aged and 10 young subjects. A significantly greater upregulation of both CD25 and CD69 antigens over the baseline levels was observed on CD19+, and CD5+ and CD5- B cells in aged humans as compared to B cells and B cell subsets in young (Table 1). Furthermore, greater upregulation was observed in CD5+ B cells as compared to CD5- B cells. When data were analyzed for the density of these molecules as measured by mean fluorescence intensity (MFI), significant increase in aging was observed for CD25 expression (Table 2).

**Table 1:** Effect of Leptin on the expression of activation markers on B cell subsets (%)

**Marker**		**Young**			**Aged**		
		**CD19**	**CD5+**	**CD5-**	**CD19**	**CD5+**	**CD5-**
CD25	Control	2.8 ± 0.4	3.5 ± 0.4	3.0 ± 0.3	3.5 ± 0.2	5.0 ± 0.7	1.8 ± 0.4
	Activated	8.3 ± 1.1	4.0 ± −.3	10.0 ± 1.3	26.3 ± 2.8	15.0 ± 2.3	12.0 ± 1.9
CD69	Control	1.8 ± 0.2	2.4 ± 0.5	1.9 ± 0.5	5.5 ± 1.0	3.1 ± 0.4	2.5 ± 0.3
	Activated	4.3 ± 0.6	2.6 ± 0.3	4.8 ± 0.3	8.2 ± 0.5	7.6 ± 0.8	6.9 ± 0.4

P value:

CD25, Aging vs. Young: CD19 = P < 0.01, CD5 + = P < 0.001, CD5- = P < 0.05.

CD69, Aging vs. Young: CD19 = P < 0.05, CD5+ = P < 0.01, CD5- = P < 0.05.

**Table 2:** Effect of Leptin on the expression of activation markers on B cell subsets (MFI)

**Marker**		**Young**			**Aged**		
		**CD19**	**CD5+**	**CD5-**	**CD19**	**CD5+**	**CD5-**
CD25	Control	3.6 ± .4	5.6 ± 2	5.6 ± 2	10 ± 4	7 ± 1.25	10 ± 7
	Activated	9.6 ± 1.7	9.5 ± 1.4	9.5 ± 1.4	26 ± 2	15 ± 2.5	51 ± 4.6
CD69	Control	4.5 ± 2	8.8 ± 5	4.1 ± 1.6	4.5 ± .4	7 ± 3.5	3.6 ± 8
	Activated	7.6 ± 2.6	11 ± 5.9	7.3 ± 1.7	8.6 ± .47	20 ± 5.7	8 ± .8

P value:

CD25, Aging vs. Young: CD19 = P < 0.01, CD5 + = P < 0.001, CD5- = P < 0.045.

CD69, Aging vs. Young: CD19 = ns, CD5+ = P < 0.02, CD5- = ns.

### Leptin-induced production of IL-6, TNF-α, and IL-10 by B cells from young and aged subjects

**Figure 2:**
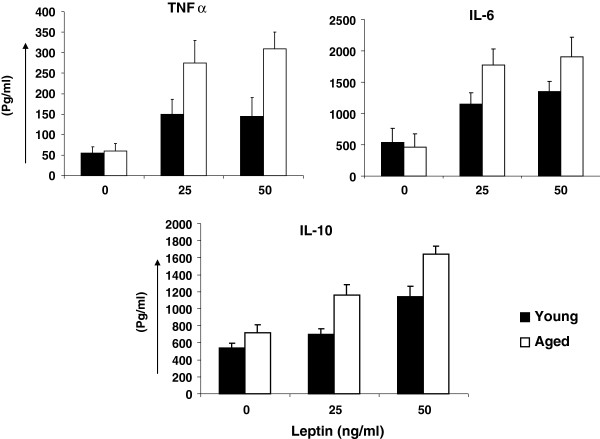
**Leptin-induced secretion of cytokines.** Purified B cells from young and aged were cultured in the absence or presence of leptin (25 ng/ml and 50 ng/ml) for 24 hours and supernatants were analyzed for TNF-α, IL-6, and IL-10 using ELISA assay. Data are expressed as mean ± sd. Each experiment was done in triplicates.

**Figure 3:**
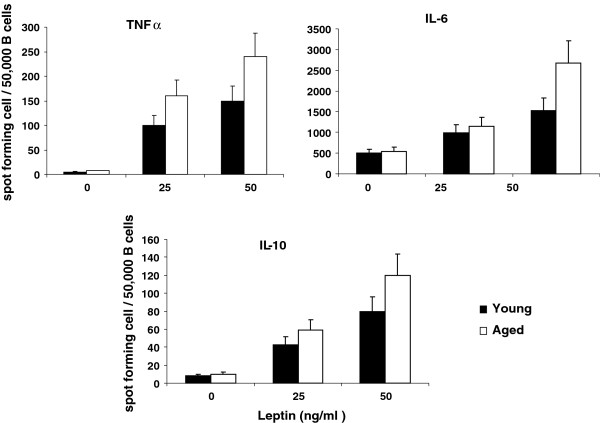
**Leptin-stimulated cytokine secreting B cells.** Purified B cells (12,500-50,000/well) from young and aged were cultured in the absence or presence of leptin (25 ng/ml and 50 ng/ml) for 24 hours and TNF-α, IL-6, and IL-10 secreting B cells were analyzed by ELISPOT assay. Data are expressed as mean ± sd. Each experiment was done in triplicates.

Purified B cells were stimulated with 25 ng/ml and 50 ng/ml of leptin for 24 hours and supernatants were collected and stored at −20°C until assayed for IL-6, TNF-α, and IL-10 by ELISA. Cytokine secreting B cells were also analyzed by ELISPOT. Figure 2 show data from ELISA assay. Leptin at both 25 ng/ml and 50 ng/ml induced significantly (P < 0.05) greater amounts of IL-6, TNF-α, and IL-10 by B cells from aged as compared to young subjects. Similar data were obtained for IL-6, TNF-α, and IL-10 secreting B cells as determined by ELISPOT assay (Figure 3). We observed no difference in these cytokines production between B cells purified by positive selection versus those purified by negative selection, therefore, excluding any possibility of activation of B cells during positive selection (data not shown).

### Increased sensitivity of B cells to leptin in aging is due to increased positive downstream signaling event

**Figure 4:**
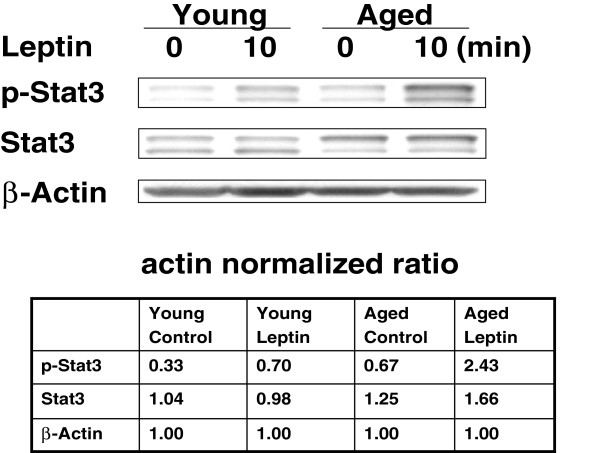
**Leptin-induced phosphorylation of STAT3 in B cells.** Purified B cells from young and aged were cutlured in the presence and absence of 50 ng/ml leptin for 10 min and native and phosphorylated STAT3 were analyzed by Western blot using specific antibodies. Actin was used an an internal loading control. Quantitation of bands was performed by densitometry and shown in table below.

Since leptin induces cytokines by activation of downstream signaling pathways, including phosphorylation of STAT3 [25], we examined leptin-induced phosphorylation of STAT3 in purified B cells from aged and young subjects. Purified B cells were stimulated with 50 ng/ml of leptin for 10 min, and phosphorylation of STAT3 was determined by Western blotting using specific antibodies. Actin was used as a loading internal control. Quantitative analysis was performed by densitometry. Figure 4 shows that leptin induces greater phosphorylation of STAT3 in aging B cells as compared to B cells from young subjects suggesting an increased activation of positive downstream signaling in aging.

## Discussion

In this study we have demonstrated that leptin activates B cells from aged humans and induces significantly higher amounts of TNF-α, IL-6, and IL-10 as compared to B cells from young subjects. This is in part due to an increased phosphorylation of STAT3.

Aging is considered a state of chronic inflammation. Plasma levels of proinflammatory cytokines, including IL-6 and TNF-α are increased in human aging [6-11]; however, the cellular source of increased proinflammatory cytokines is poorly understood. These cytokines are produced by T cells, macrophages, and B cells. However, macrophage-derived IL-1β, TNF-α and IL-6 and Th1 derived cytokines are decreased in aging [16-20]. Therefore, B cells is likely one of the sources of increased proinflammatory cytokines associated with human aging, though dendritic cells in aging may also contribute to increased levels of proinflammatory cytokines [26]. Recently, B cells have shown to produce a number of cytokines, chemokines, and growth factors with proinflammatory properties [21-25].

Leptin is one of adipokines, which is powerful inducer of inflammatory cytokines by Th1 T cells and macrophages, including IFN-γ, IL-1β, TNF-α, and IL-6 [1-5,27,28]. More recently, we have reported that leptin also activates human B cells to induce inflammatory cytokines, including IL-6 and TNF-α, and anti-inflammatory/ immunoregulatory cytokine IL-10 [29-31]. We have reported that leptin activates human B cells [25], and serum levels of leptin are increased in aging [17-20]. Since leptin and proinflammatory cytokines are increased in aging, and T cell-and macrophage-derived proinflammatory cytokines are decreased in aging, we investigated the effects of leptin on purified B cells. In the present study we show that leptin activates human aged B cells to a greater extent than B cells from young subjects as evidenced by upregulation of CD25 and CD69, which was observed on both CD19 + CD5+ B cells and CD19 + CD5-B cells, greater upregulation was observed in CD5+ B cells as compared to CD5- B cells. CD5+ B cells are reported to produce IL-10 with immunoregulatory property [32], which may explain a role of leptin in autoimmunity [1,33].

Activation of B cells was also associated with increased production of cytokines by B cells. Leptin induced significantly higher (P < 0.05) secretion of IL-6, TNF-α, and IL-10 by B cells (ELISA assay) from aged humans as compared to B cells from young subjects. Furthermore, we demonstrate that leptin not only induced increased amounts of secreted cytokines by aged B cells, but leptin also increased significantly higher number B cells (ELISPOT assay) to secrete these cytokines. Increase in leptin-induced IL-10 in aging may suggest a compensatory mechanism to counterbalance increased IL-6 and TNF-α production. Since B regulatory cells produce IL-10 [34] it is possible that increased IL-10 production might be due to increased Breg in aging. This possibility is under investigation. IL-10 rescues CD5+ cells from apoptosis and augments the proliferation of CD5- B cells [35-39]. Since IL-6 and TNF-α are produced by both CD5+ and CD5- B cells, increased leptin-induced B cell-derived IL-10 in aging may be playing a role in aging-associated chronic inflammation by surviving B cells from apoptosis to produce IL-6 and TNF-α. Buffa et al. [40] have reported an expansion of CD19 + CD38-CD24- B cells in aged humans and these cells produce both TNF-α and IL-10.

Leptin signal via three distinct pathways, including JAK-STAT, PI3K, and MAPK pathways [41-43]. Following binding of leptin with leptin receptor, JAK2 (Janus kinase 2) is activated by auto-or cross phosphorylation. Activated JAK2 tyrosine phosphorylates intracellular domain of leptin receptor. The phosphorylated receptor provides a docking site for STAT (signal transducer and activator of transcription) factors, particularly STAT3, which is substrate of JAK2. STAT3 itself is a substrate for JAK2 and homo- or hetero-dimerization upon phosphorylation, translocates to the nucleus, and modulates transcription of genes, including cytokine genes. We show that Leptin induced significantly greater phosphorylation of STAT3 in B cells from aged subjects as compared to B cells from young controls, suggesting that leptin-induced increased cytokine production by aged B cells is via increased activation of STAT3.

## Conclusions

Leptin activates B cells from aged humans via increased intracellular signaling to secrete IL-6, TNF-α, and IL-10 to a greater extent than B cells in young subjects, which may contribute to chronic inflammation associated with human aging.

## Materials and methods

### Subjects

Peripheral blood mononuclear cells (MNCs) were isolated from blood of 10 healthy young (18–35 years, mean 23±4) and 10 healthy aged (66–84 years, mean 74±6) subjects by Ficoll–hypaque density gradient. Aged subjects are healthy people of middle and upper middle social status, and are living active independent lives in the city of Laguna Woods, California (senior community). All subjects were required to discontinue all vitamins and antioxidants (if they were taking) for at least one year prior to donation of blood sample. Protocol was approved by Human Subject Committee of the Institution Review Board of the University of California, Irvine. Consent form approved by the Institutional Review Board of the University of California was signed by each subject. Consent form states that publications and/or presentations that result from this study will not include identifiable information about you.

### Antibodies and reagents

The following anti-human immunoglobulins were used: CD19 PerCP, CD69 FITC, CD25 APC, and CD5 PE; all from BD Parmingen (San Jose, CA). B cell enrichment kit was purchased from Stem cell Technologies (Vancouver, BC, Canada), and recombinant human leptin from PeproTech (Rocky Hill, New Jersey).

### Immunophenotyping

B cells were stained with, APC-conjugated anti-CD25, FITC-conjugated anti-CD69, PE-conjugated anti-CD5 monoclonal antibodies. After staining, cells were washed extensively with phosphate-buffered saline and analyzed by Flow cytometry using FACScalibur (Becton-Dickenson, San Jose, CA) equipped with argon ion laser emitting at 488 nm (for FITC, PE, and PerCP excitation) and a spatially separate diode laser emitting at 631 nm (for APC excitation). Forward and side scatters were used to gate and exclude cellular debris. Ten thousand cells were acquired and analyzed using Flowjo software (Treestar, Ashland, OR). Data were analyzed for both percentages of cells expressing CD25 and CD69 as well as density of molecules as defined by mean fluoresence intensity (MFI).

### Purification of B cells

B lymphocytes were purified by immunomagnetic human B cell enrichment kit according to manufacturer’s instructions (STEMCELL Technologies, Vancouver, Canada). In brief, peripheral blood mononuclear cells were suspended at no more than 1 × 10^8^ cells/ml in PBS containing 2% FBS. Negative selection cocktail (100 μl/ml) was added and incubated at room temperature for 15 min. The magnetic nanoparticles were added at 50 μl/ml cells and incubated for 10 min. Cells were placed in a 12 × 75-mm polystyrene tube at a volume of 2.5 ml/tube and placed into the magnet for 5 min. The magnet was inverted, and the supernatant was poured off. The magnetically labeled unwanted cells remain bound inside the original tube. The purity of negatively selected cells was assessed by flow cytometry (>97%) as detected by the presence of CD20.

### Measurement of cytokines

Cytokine secretion was measured by ELISA, and ELISPOT assays. Purified B cells were activated by 25 ng/ml and 50 ng/ml of leptin for 24 h. Supernatants were collected and stored at −20°C until assayed for detection of cytokines by ELISA (ELISA kits from BD Pharmingen, San Jose, CA) as per manufacturer’s protocol. Cytokine secreting cells were measured by ELISPOT assay according to manufacturer’s protocol. In brief, PVDF membrane ELISPOT plates (Millipore, Billerica, MA) were coated with 100 μl (conc. 2 μg/ml) of capture antibody per well (BD Pharmingen, San Jose), and were kept overnight at 4°C. Plates were washed 3 times with sterile PBS and 200 μl/well of sterile blocking buffer was added for ≥ 1 hour. Plates were washed 3 times with sterile PBS. Purified B cells (12,500; 25,000; and 50,000/well) were placed in triplicate. Twenty five and 50 ng/ml leptin was added to each well and plates were incubated at 37°C 5% CO_2_ incubator for 48 hrs. Plates were first washed x3 times with PBS, and then 3 times with PBS-Tween. 100 μl/well of diluted biotinylated detection antibody (BD Pharmingen, San Jose, California) was added at 2 μg/ml and incubate for 2 hr at room temperature. Plates were washed with PBS-Tween, and 100 μl/ well of the Av-HRP conjugate (BD Pharmingen, San Jose, California) added and incubate at room temperature for 1 hours. 200 μl/well of fresh substrate solution was added and spot/color development was monitored. Reaction was stopped by rinsing plate with tap water, and followed by blotting on paper towels. Plates were allowed to air dry overnight. Spots were counted manually with a dissecting microscope, and expressed as spots/50,000 B cells.

### Western blotting

Purified B cells were incubated in the presence or absence of 50 ng/ml of leptin for 10 min, and cells were lysed with lysis buffer. Aliquots of cell lysates containing 20 μg of total protein were resolved by SDS–PAGE and transferred onto membranes (Millipore, Bedford, MA) by electroblotting. The membranes were blocked for 1 h at room temperature in TBS-T buffer with 5% nonfat dried milk and incubated with primary antibodies overnight at 4°C. The blots were washed three times for 20 min with TBS-T buffer and then incubated with HRP-conjugated secondary antibodies (1:3,000–1:6,000 dilution) for 1 h at room temperature. After washing three times for 20 min in TBS-T buffer, blots were developed using enhanced chemiluminescence reagents (ECL, Pierce Biotechnology, Inc., Rockford, IL) and exposed to clear-blue X-Ray film. Actin was used as a loading control. Bands were scanned, and volumes were calculated. Quantification was done by a ratio between STAT-3 or pSTAT-3 and actin.

Statistical analysis was performed by paired student t test

## Competing interests

The authors declare that they have no competing interests.

## Authors’ contributions

SG made the hypothesis, designed the experimental approach, and written the manuscript. SA performed cytokine ELISA and ELISSPOT assays for cytokines. SG set-up the cultures and did the flow cytometry for activation markers. Manuscript was read and edited by all authors.
